# Dynamic modulation of spatial selection: Online and anticipatory adjustments in the flanker task

**DOI:** 10.3758/s13414-025-03026-5

**Published:** 2025-02-20

**Authors:** Mercedes B. Villalonga, Abigail L. Noyce, Robert Sekuler

**Affiliations:** 1 Department of Psychology, Brandeis University, 415 South Street MS 062, Waltham, MA 02453 USA; 2 Neuroscience Institute, Carnegie Mellon University, Pittsburgh, PA USA; 3 Neuroscience Program, Brandeis University, Waltham, MA USA

## Abstract

To track the spatiotemporal dynamics of selective attention, we constructed four theory-driven variants of Eriksen’s flanker task. In each, subjects made speeded binary categorizations of target arrowhead direction while ignoring surrounding flanker arrowheads, whose direction was either congruent or incongruent to the target. Experiment 1 tracked the temporal evolution of target selection by systematically manipulating onset asynchrony between the target and flankers. In Experiments 2A and 2B, we increased flanker strength (both experiments) and reduced target strength (Experiment 2B only) at various times relative to target onset, exploring the effects of dynamic perceptual inputs on flanker congruency effects. Experiment 3 measured how uncertainty about stimulus location impeded spatial selection. Our findings demonstrate that spatial selection in the flanker task is dynamically modulated by both intra- and supra-trial factors.

**Supplementary Information:**

The online version contains supplementary material available at 10.3758/s13414-025-03026-5.

## Introduction

Vision utilizes spatial selection to scrutinize goal-relevant regions of space while managing limited processing resources (Carrasco, 2011). One influential account of spatial selection incorporates the metaphor of a *zoom lens* (Eriksen & St. James, 1986). A camera’s zoom lens affords dynamic control over the field of view. This feature has been invoked to describe the dynamic mechanisms that serve visual spatial selection (Braver, 2012; Nobre & van Ede, 2023), such as the trade-off between an attended region’s size and processing efficiency within that region (Feldmann-Wüstefeld & Awh, 2020; Eriksen & Schultz, 1979; Eriksen & St. James, 1986; Müller et al., 2003). In unpredictably dynamic environments, a photographer must adjust their zoom lens in real time while also aiming their camera. Experimental evaluations of such *reactive* adjustments of visual selection focus on within-trial, stimulus-driven dynamics (White et al., 2011, 2018; Evans & Servant, 2020; Weichart et al., 2020; Kobayashi et al., 2022). Alternatively, a photographer might be able to anticipate what zoom setting a situation will require. In a vision experiment, such anticipatory, *proactive* adjustment of spatial selection might be evoked in response to some pre-trial cue or over the course of multiple trials (Botvinick et al., 2004; Bulger et al., 2021; Koob et al., 2023; Noyce & Sekuler, 2014). These two perspectives (real-time, within-trial adjustments and anticipatory, between-trial adjustments) are complementary, but each alone is incomplete. Additionally, studies investigating reactive adjustments of spatial selection have only used static stimuli (Hübner et al., 2010; Ulrich et al., 2015; White et al., 2011). In the real world, vision must respond to time varying inputs, but little is known about how these dynamics impact the selection process.

Eriksen’s flanker task is a simple behavioral paradigm whose flexibility enables a nuanced dissection of spatial selection (Eriksen & Eriksen, 1974). Subjects make a speeded binary judgment about the identity of a single target stimulus surrounded by several salient but task-irrelevant distractors. Subjects tend to make fewer errors and respond faster when the target and flanking distractors are congruent than when they are incongruent. Known as the *flanker congruency effect* (FCE), this difference in performance indexes the efficacy of visual selection. In the current study, we used the flanker task to examine spatial selection across multiple time scales simultaneously, bridging within-trial and between-trial perspectives.

### Within-trial adjustments of selection

Both behavioral and neurophysiological studies have demonstrated reactive, within-trial modulations of spatial selection in response to conflict in the flanker task (Jia et al., 2024; Mittelstädt et al., 2022, 2023; Nigbur et al., 2015; Scherbaum et al., 2011; Weichart et al., 2020). The temporal relationship between flanker interference and target processing has been evaluated via sequential sampling models (SSMs) of spatial selection (Hübner et al., 2010; Hübner & Töbel, 2019; Mackenzie et al., 2022; Ulrich et al., 2015; White et al., 2011) as well as distributional response time analysis (Mackenzie et al., 2022; Mittelstädt et al., 2022, 2023; Pratte, 2021; Ulrich et al., 2015). The mechanistic assumptions of SSMs successfully capture behavior under various flanker task conditions and reveal within-trial modulation of selection (Servant & Evans, 2020; White et al., 2018). Weichart et al. (2020), for example, found that an evidence accumulator with a high degree of within-trial variability provided the best model fit, suggesting the spatial extent of the “zoom lens” varies not just between trials but also *within* each trial. Further, distributional response time analysis suggests that selection is dynamically modulated by an interference process during each flanker task trial. Both active suppression and passive decay of flanker interference have been suggested as potential mechanisms governing the within-trial variability of selection efficacy (Ridderinkhof, 2002; Ulrich et al., 2015).

These studies provide evidence of reactive, within-trial modulations of spatial selection, but their scope has been limited. For example, most studies focusing on SSM analyses make comparisons between different conflict tasks (e.g., flanker, Simon, and Stroop tasks: Hübner & Töbel, 2019; Mackenzie et al., 2022; Pratte et al., 2010) or between conditions using stimuli processed at different levels of the visual hierarchy (Pratte, 2021). A notable limitation of previous investigations is the predominant use of static visual stimuli (Hübner et al., 2010; Ulrich et al., 2015; White et al., 2011). Stimuli with dynamic visual features (e.g., mid-trial changes in luminance, size, or location) model real-world situations requiring spatial selection with greater accuracy than static stimuli. Studies of visual motion processing suggest that the flanker task engages a unified selection mechanism shared across processing streams in the visual cortex (Katzner et al., 2009; Lange-Malecki & Treue, 2012; Treue & Martinez-Trujillo, 2012). However, no study has evaluated within-trial selection processes in the flanker task by using stationary stimuli with dynamic visual features.

### Anticipatory adjustments of selection between trials

Spatial selection is also subject to modulatory influences on timescales extending *beyond* a single trial. Modulation of selection can occur from one trial to the next (*sequential* or *carry-over* effects) as well as between blocked conditions (*block-wise* or *global* effects, Bulger et al., 2021; Bugg & Crump, 2012; Nigbur et al., 2015; Noyce and Sekuler, 2014). Effects on the shorter timescale have been more widely researched: for example, the FCE on a trial *N* is often smaller when following an incongruent trial $N-1$, compared to a congruent trial $N-1$ (the *congruency sequence effect*; Coles et al., 1985; Gratton et al., 1992; see Egner, 2007 for a review). Smaller FCEs are also observed in blocks that contain mostly incongruent trials (the *proportion congruent effect*; see Bugg & Crump, 2012 and Schmidt, 2019 for reviews). Proportion congruent effects occur regardless of trial order, indicating a global modulation of spatial selection that occurs over a longer time scale than that yielding congruency sequence effects.

Neurophysiological evidence suggests that proactive, between-trial adjustments of spatial selection comprise a form of cognitive control and are a response to the level of uncertainty about upcoming trial conditions (Cavanagh & Frank, 2014). Multiple investigations have explored how different forms of uncertainty impact spatial selection within the framework of the flanker task. For example, the proportion of congruent/incongruent trials, as described above, is one manipulation of uncertainty: increased incongruent trial frequency creates regularity and begets conflict adaptation (Bugg & Crump, 2012). In another study, researchers manipulated the probabilities of different flanker types: and stimuli occurred more frequently than and (Bulger et al., 2021; Noyce & Sekuler, 2014). Less frequent “oddball” stimuli disrupted performance and produced larger FCEs, even while controlling for proportion congruent and response likelihood. These and other studies suggest that uncertainty about stimulus probability, an expectation formed as the result of experience across numerous trials, modulates spatial selection in flanker tasks (Asanowicz et al., 2023; Donohue et al., 2016; Kerns et al., 2022).

**Table 1:** Subject information for all experiments

Experiment	n	Mean age (years)	Gender	Mean Snellen acuity$^{\text {a}}$
1	40$^{\text {b}}$	18.7	33 women, 7 men	20/16.7
2A	23	19.2	14 women, 9 men	20/17.1
2B	23$^{\text {b}}$	19.2	16 women, 7 men	20/16.9
3	22	19.3	12 women, 10 men	20/17.9

$^{\text {a}}$Visual acuity was measured with Sloan ETDRS 2000 Series chart at 60 cm viewing distance

$^{\text {b}}$One subject who participated in Experiment 1 also participated in Experiment 2B

### The current study

Our objectives were to investigate within-trial and between-trial modulations of spatial selection simultaneously in the flanker task. To provide a coherent framework for examining both reactive and proactive adjustments, we standardized key variables: stimulus materials (Pratte, 2021), speed-accuracy instructions (Mittelstädt et al., 2022), the proportions of congruent and incongruent stimuli (Wendt & Luna-Rodriguez, 2009), and the timing of successive trials (Sussman & Sekuler, 2022). Experiment 1 investigated the temporal dynamics of spatial selective attention by manipulating target-flanker stimulus onset asynchrony (SOA) and analyzing the FCE across response time distributions. We hypothesized that variable amounts of flanker interference generated by different SOAs would lead to observable variations in the time course of spatial selection. We further tracked the within-trial time course of selection in Experiments 2A and 2B, where we explored the effects of within-trial dynamic perceptual inputs. We tested the limits of spatial selection processes by boosting flanker interference at different trial time points, expecting the earliest boosts to disrupt selection most, a hypothesis suggested by our findings from Experiment 1. In Experiment 3, we compared the relative contributions of spatial uncertainty on selection across two different timescales: both at the local level of trial sequence, as well as at a global (block) level.

## Experiment 1

To track spatial selection’s temporal evolution, this experiment manipulated the onset asynchrony between the target and flankers. Extending previous work (Eriksen & Schultz, 1979; Flowers & Wilcox, 1982; Flowers, 1980; Hübner & Töbel, 2019; Mattler, 2003; Mackenzie et al., 2022), we measured the FCE at nine different SOA values, all within a sub-second range. We hypothesized this narrow time window would bracket the rapid evolution of the FCE. After comparing mean flanker effects at various SOAs, we examined the temporal dynamics operating within each SOA condition in greater detail.

**Fig. 1:**
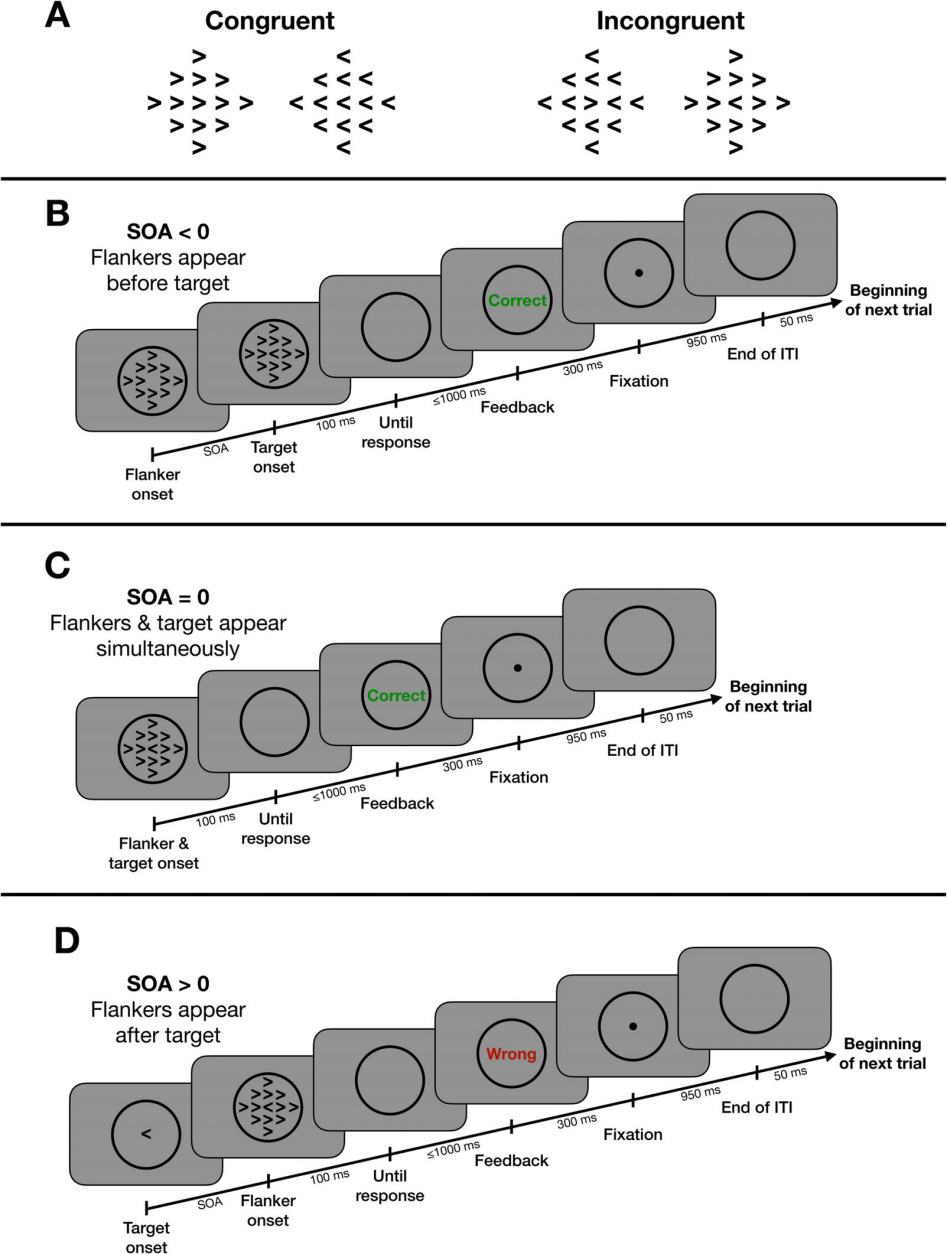
(**A**) The stimulus arrays used in Experiment 1. Target direction (left and right) and flanker congruency (Congruent and Incongruent) were fully balanced; all four arrays were presented with equal frequency. (**B-D**) The series of events within one trial when the flankers’ onset preceded (**B**), co-occurred with (**C**), or followed (**D**) the onset of the target. ITI: inter-trial interval. SOA: stimulus onset asynchrony

We expected our SOA manipulation to impact the within-trial time course of selection and interference processes (Eriksen & Schultz, 1979; Flowers & Wilcox, 1982; Flowers, 1980; Hübner & Töbel, 2019; Mattler, 2003; Pratte, 2021; Ridderinkhof et al., 2005; Wylie et al., 2007, 2009). In conditions when flankers preceded the target, we reasoned that flankers would generate a strong directional signal before the target appeared. We expected a maximal FCE on these trials, reflecting the increased degree of flanker signal inhibition required for accurate target selection. In conditions when flankers appeared simultaneously with or after the target, spatial selection could proceed with less (or no) flanker interference. In these conditions, the flankers’ influence would lag behind target processing, which we expected to result in smaller FCEs.

### Method

#### Subjects

We collected data from 40 Brandeis University undergraduates. Our sample size gave us >95% power to detect the smallest congruency effect size reported in a similar recent study ($$\eta _p^2 =.61$$; Mackenzie et al., 2022). Subjects were either paid $15 USD (n=15) or received course credit (n=25) for participation. Table 1 gives subject demographic information.

#### Apparatus & stimulus

Subjects viewed the stimulus display from a distance of 60 cm. The display was a 54.6-cm computer monitor (Dell U2211H), with 1920 x 1080 pixel resolution and 60 Hz refresh rate. Stimuli were presented using PsychoPy Version 2021.2.3 (Peirce et al., 2019), running on a Mac Mini (Mid 2011, macOS Sierra, Version 10.12.6). Subjects communicated their responses using a standard QWERTY keyboard, as described below.

Following Weichart and Sederberg (2021), we created a flanker array in which the target was surrounded by 12 arrowheads (Fig. 1). Each arrowhead (12.8 cd/m^2^ luminance) subtended 1° $\times$ 1° visual angle, with neighboring arrowheads separated by 1.25° center-to-center. In Congruent stimulus arrays, the target and flankers pointed in the same direction, either rightward or leftward; in Incongruent stimulus arrays, the target and flankers pointed in opposite directions: a rightward target arrowhead was surrounded by leftward flankers, or *vice versa*. Presented on a mid-gray background (48.4 cd/m^2^), stimulus arrays were centered within a thin black circle (8° diameter) that remained on the screen between trials to encourage fixation (Fig. 1).

Flanker-target SOA varied randomly from trial to trial, with flankers appearing [-400, -200, -100, -50, 0, +30, +50, +100, +200] ms relative to the target. Negative SOA values indicate conditions in which flanker onset *preceded* target onset; positive SOA values indicate flanker onset *followed* target onset; at SOA = 0 ms, the onsets of flankers and target were simultaneous. For each SOA, all 12 flankers appeared simultaneously, and as mentioned above, all were pointed in the same direction, either left or right.

#### Task

Figure 1 shows examples of trial timelines. The entire stimulus array remained on the screen for 100 ms after the scheduled SOA in all conditions. Subjects were required to respond within 1 s of stimulus offset. Subjects pressed either the ’f’ key with their left index finger or ’j’ key with their right index finger to indicate a or target arrowhead, respectively. Immediately after a response, feedback (described below) was given for 300 ms. Then, subjects fixated on a small black disc (0.5° diameter) for 950 ms. The fixation point disappeared 50 ms before the next stimulus appeared (Fig. 1).

Subjects completed eight experimental blocks of 108 trials, each with an equal frequency of the four possible display configurations: the two directions of target orientation crossed with the two levels of Congruency. During each block, these four combinations were presented three times at each SOA, in random order. This yielded 864 trials per subject. Before the first experimental block, subjects completed a practice block of 36 trials, which included one of every trial type. If a subject scored <70% correct on the first practice block, they repeated the practice block to make sure they understood the task. Only two subjects had to repeat the practice block; these subjects made a disproportionate number of errors on the first practice block because they tended to respond prematurely on trials with SOA < 0 (i.e., they responded before the target appeared). Both of these subjects achieved >70% correct on their second practice block.

Subjects received feedback following each trial and block. After each trial with a correct response, the word “CORRECT” was shown in green accompanied by a pleasant chime sound; after each incorrect, missing, or premature (*i.e*., occurring before the target appeared) response, the word “WRONG” was shown in red accompanied by a mildly unpleasant buzzer sound (Fig. 1). After each block, additional feedback was given as a composite performance score that was designed to encourage subjects to give equal importance to speed and accuracy. Following Weichart and Sederberg (2021), we calculated composite scores using Eqs. 1-3:

1 $$\begin{aligned}&\text {speed} = \frac{\sum \limits _{i \in I} \frac{\ln (3000) - \ln (i + 1000)}{\ln (3000) - \ln (1300)}}{N_{\text {total}}} \end{aligned}$$

2 $$\begin{aligned}&\text {accuracy} = \frac{N_{\text {correct}}}{N_{\text {total}}} \end{aligned}$$

3 $$\begin{aligned}&\text {score} = \frac{\text {accuracy} - 0.5}{0.5} \times \text {speed} \times 100 \end{aligned}$$

where *i* is the response time (in milliseconds) on each trial in the set *I* of that block’s RTs. Scores ranged between 0 and 100.

Between blocks, the subject received their score for the last block, as well as a reminder of their highest score so far and instructions for how to earn higher scores: "To improve your score, try to respond as fast as you can without making errors.” To engage subjects and to encourage sustained levels of performance throughout the task, upon entering the laboratory, each subject was shown a leader board that displayed the ten highest scores along with the initials of previous subjects and was encouraged to aim for a score in that range.

#### Data analysis

For each trial, we recorded accuracy and response time (RT) in milliseconds relative to the time of target onset. We referenced RT relative to target onset rather than the onset of the entire trial because the time of target onset varied with SOA. We calculated the mean error rate (ER) and median correct RT for each subject’s 18 (SOA$\times$Congruency) conditions. For the RT analysis, trials with an RT < 200 ms were considered incorrect. We analyzed ER and RT using separate two-way (SOA$\times$Congruency) within-subject ANOVAs. We then calculated each subject’s mean FCE as the difference between performance on Congruent and Incongruent trials, for each dependent variable. Error rate flanker congruency effect (FCE$_{ER}$) and response time flanker congruency effect (FCE$_{RT}$) were calculated in each of the nine SOA conditions for each subject.

**Fig. 2:**
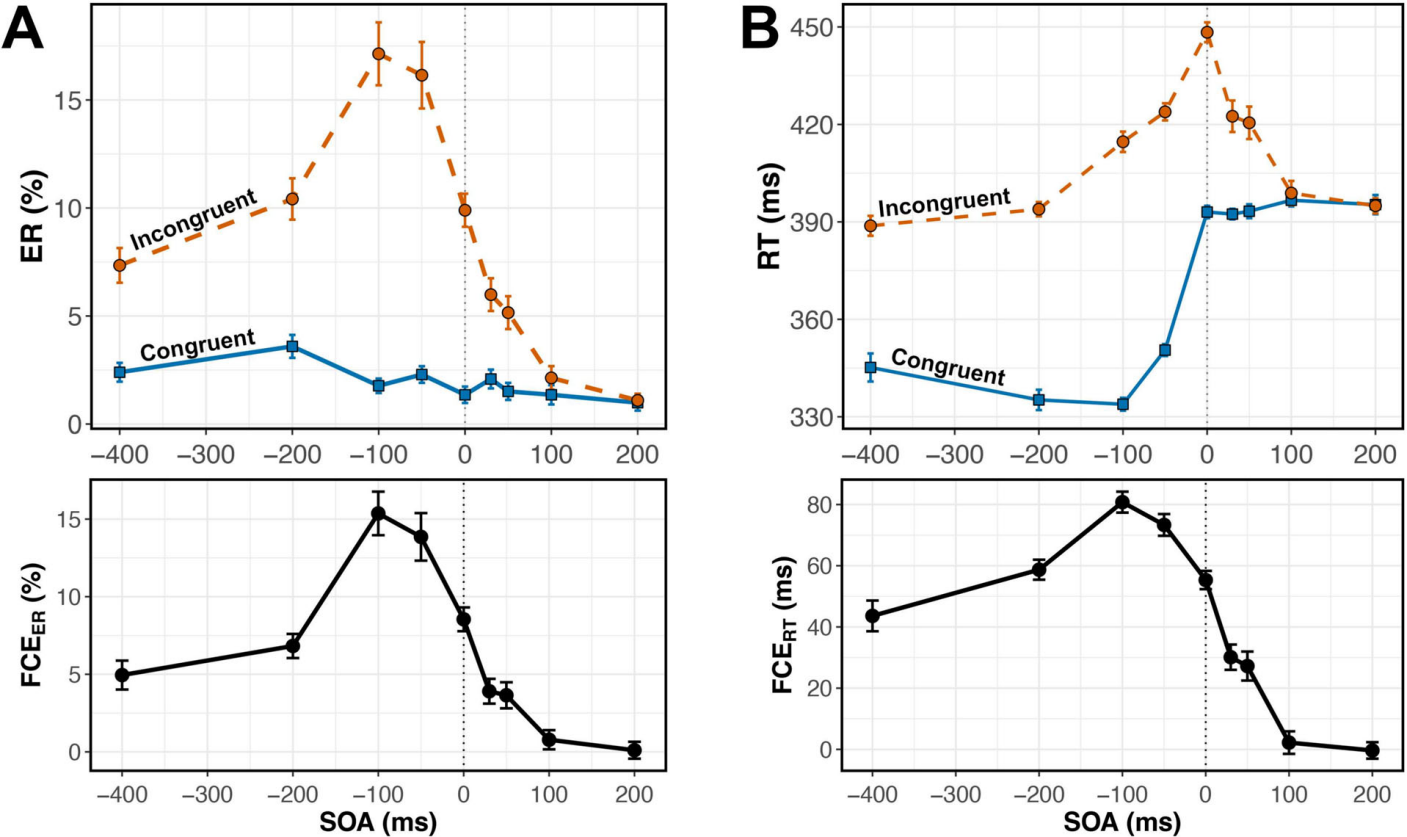
(**A**, top) Error rates were lower on Congruent (blue, solid) than Incongruent (orange, dashed) trials across SOAs, and the effect of SOA manifested primarily in Incongruent trials. (**A**, bottom) The FCE$_{ER}$ was largest when flankers preceded the target by 100 ms or less. (**B**, top) RT was shorter on Congruent than Incongruent trials across SOAs, with SOA effects appearing on both Congruent and Incongruent trials. (**B**, bottom) The FCE$_{RT}$ was again largest when flankers preceded the target by 100 ms or less. Error bars reflect within-subject standard error, n=40

For a more detailed analysis of temporal effects, we analyzed FCE$_{RT}$ magnitude at different quantiles of the RT distribution (a delta plot; De Jong et al., 1994) for each SOA condition. A delta plot’s slope reflects the relative time courses of target and flanker processing (Mackenzie et al., 2022; Pratte, 2021; Ulrich et al., 2015). When FCEs are larger at long RTs than at short RTs (positive delta plot slope), it implies that flanker information accumulates and diminishes over a longer time course than target information. Conversely, when FCEs are larger at short RTs (negative delta plot slope), it implies that flanker information accumulates and diminishes faster than target information. Non-monotonic delta plots may additionally reflect the time course of cognitive control mechanisms (e.g., the onset of suppression; Pratte, 2021; Ridderinkhof, 2002; Ridderinkhof et al., 2005).

We generated delta plots using the established procedure (De Jong et al., 1994). We first sorted each subject’s RTs (correct trials only) in ascending order and calculated nine percentile interval values (10, 20,... 80, 90%), separately for each of the 18 conditions (SOA $\times$ Congruency). We performed the following procedures for each SOA condition and each subject: First, we averaged the subject’s Congruent and Incongruent RTs at each percentile, generating nine RT values, each representing a single bin of the subject’s overall RT distribution. Next, for each percentile bin, we subtracted the mean Congruent RT from the mean Incongruent RT, yielding the FCE$_{RT}$ for that percentile.

We then fit a linear regression model to the relationship between percentile RT and FCE$_{RT}$ for each subject and each SOA. Separately for each SOA, we tested whether the mean delta plot slope (averaged across subjects) differed from zero. To visualize group-level delta plots across SOAs, we calculated the group-level mean RT for each of the nine percentile bins to plot along the x-axis. We then plotted the group-level mean FCE$_{RT}$ along the y-axis as a function of the RT percentile, producing one delta plot for each of the nine SOA conditions.

### Results

Before analyzing the effect of SOA and Congruency on our dependent measures (error rate and response time), we checked for any difference in performance between subjects who participated for course credit and those who were paid. We ran separate ANOVAs that included compensation group (paid vs. course credit) as a between-subjects factor. As shown in Supplementary Table 1, results from these analyses indicated that neither mean ER nor mean RT differed between groups, and no interactions that included the group term were significant. We thus combined the groups in all analyses.

**Fig. 3:**
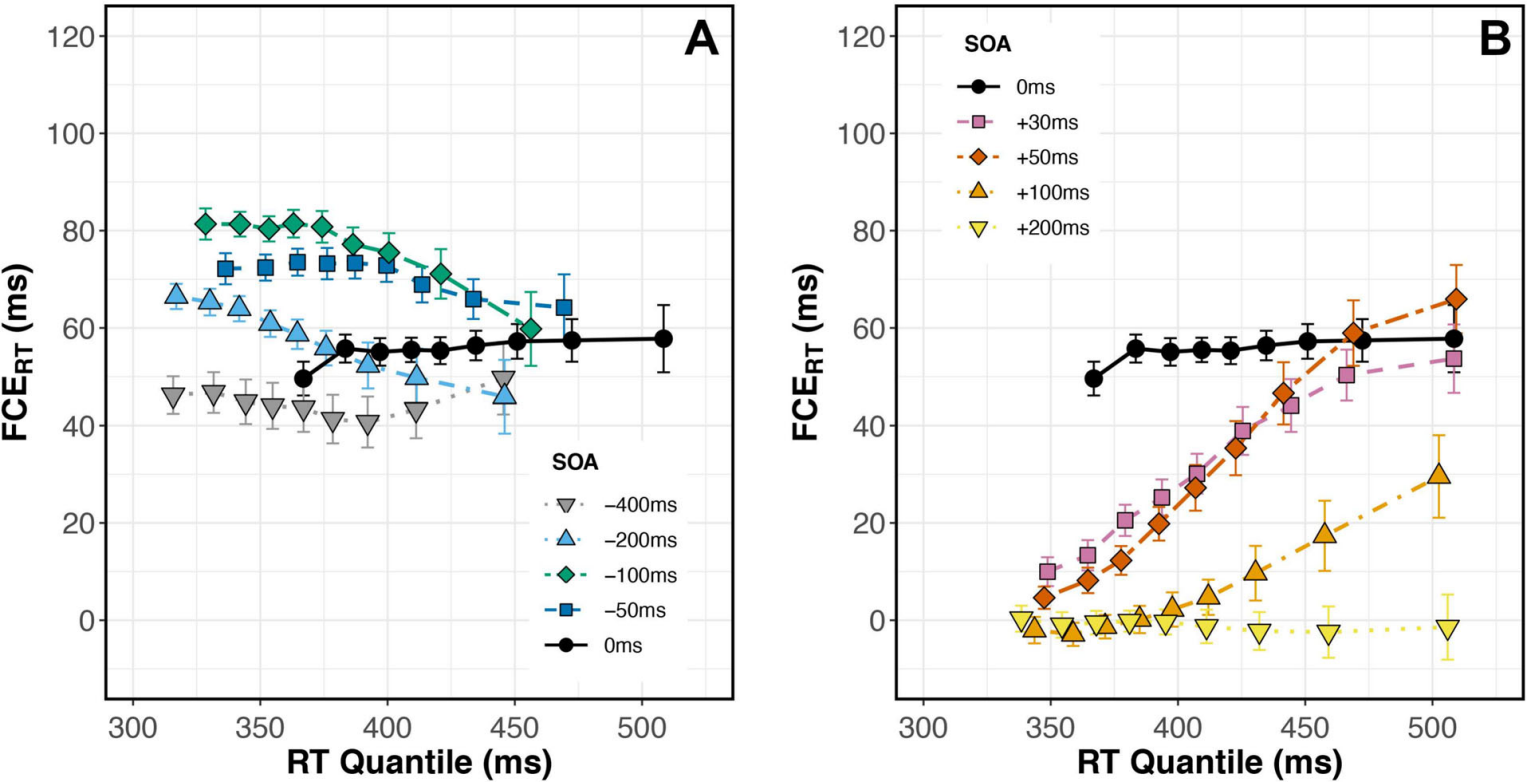
(**A**) Group average delta plots for all negative SOAs and SOA 0 ms. At SOAs of –200 (light blue triangles) and –100 ms (green diamonds), the slope across RT quantiles was significantly negative. (**B**) Group average delta plots for all positive SOAs and SOA 0 ms. At SOAs of 30 (pink squares), 50 (red diamonds), and 100 ms (orange triangles), the slope across RT quantiles was significantly positive. Flankers were processed faster than targets when they preceded by 100-200 ms, and more slowly when they followed targets by 30-100 ms

#### Error rate

Mean ER was lower on Congruent than Incongruent trials (Fig. 2A). Within-subject ANOVA showed a significant main effect of Congruency, $F(1, 39) = 170.6$, $p <.001$, $\eta _p^2 =.814$. Pairwise comparisons analyzing the simple effect of Congruency revealed a significant FCE$_{ER}$ ($p <.001$) at all SOAs except +100ms and +200ms ($p =.096$ and $p =.767$, respectively). Error rate also had a significant main effect of SOA, $F(3.24, 126.18) = 32.87$, $p <.001$, $\eta _p^2 =.457$ (adjusted for sphericity, Greenhouse-Geisser $\varepsilon =.404$).

We observed a significant Congruency $\times$ SOA interaction, suggesting that FCE$_{ER}$ magnitude varied with SOA, $F(3.06, 119.18) = 30.51$, $p <.001$, $\eta _p^2 =.439$ ($\varepsilon =.382$). Varying the SOA affected error rates on Incongruent but not Congruent trials (Fig. 2A). The largest FCE$_{ER}$ was produced when flankers appeared 50-100 ms before the target, that is, in the –50ms and –100ms SOA conditions (Fig. 2A). FCE$_{ER}$ at these SOAs were significantly greater than those at all other SOAs (Supp. Table 2). When flankers were presented $\ge$ 100 ms after the target, FCE$_{ER}$ was virtually zero.

#### Response time

Median RT on correct trials was lower on Congruent than Incongruent trials (Fig. 2B). Within-subject ANOVA showed a significant main effect of Congruency, $F(1,39) = 446.15$, $p <.001$, $\eta _p^2 =.920$. As with error rates, pairwise comparisons analyzing the simple effect of Congruency showed a significant FCE$_{RT}$ ($p <.001$) in all SOA conditions except for the +100ms and +200ms SOA conditions ($p =.553$ and $p =.902$, respectively). There was also a significant main effect of SOA, $F(3.34, 130.36) = 73.10$, $p <.001$, $\eta _p^2 =.652$ ($\varepsilon =.418$). Presenting flankers before the target sped up responding on both Congruent *and* Incongruent trials, but disproportionately more for Congruent trials, as evidenced by a statistically significant Congruency $\times$ SOA interaction, $F(4.73, 184.55) = 58.10$, $p <.001$, $\eta _p^2 =.598$ ($\varepsilon =.592$). As with error rates, the largest FCE$_{RT}$ was produced when flankers appeared 50-100 ms before the target (Fig. 2B, Supp. Table 2).

#### Time course of FCE

We examined the time course of the FCE$_{RT}$ in each SOA condition (Fig. 3). When flanker onset preceded target onset by 100 or 200 ms, delta plot slopes were significantly less than zero (Fig. 3A; 100 ms SOA: $t(39) = -2.36$, $p =.042$, $d = -.37$; 200 ms SOA: $t(39) = -2.57$, $p =.032$, $d = -.41$; Benjamini and Hochberg (1995) corrected for nine comparisons). At these SOAs, flanker information appears to be processed faster than target information. When flanker onset followed target onset by 30, 50, or 100 ms, delta plot slopes were significantly positive (Fig. 3B; 30 ms SOA: $t(39) = 6.81$, $p <.001$, $d = 1.08$; 50 ms SOA: $t(39) = 9.14$, $p <.001$, $d = 1.45$; 100 ms SOA: $t(39) = 3.39$, $p =.005$, $d =.54$; Benjamini and Hochberg (1995) corrected for nine comparisons). At these SOAs, target information appears to be processed faster than flanker information. At all other SOAs (–400, –50, 0, and 200 ms) delta plot slopes did not significantly differ from zero ($p >.2$ after Benjamini and Hochberg (1995) correction).

### Discussion

As SOA varied from –400 to –50 ms, both error rate and response time FCEs increased, reaching a maximum at –100 to –50 ms. The FCE then declined steadily to zero by SOAs of +100 ms and beyond. Interestingly, the maximal FCE did not occur at a 0ms SOA (Fig. 2), the condition most frequently used in experiments with the flanker task.

Trials with flankers preceding targets by 50-100 ms produced the largest flanker effect, consistent with previous studies (Eriksen & Schultz, 1979; Flowers & Wilcox, 1982; Hübner & Töbel, 2019; Mackenzie et al., 2022; Mattler, 2003; Wendt et al., 2014). Our study went further, dissecting the time course of selection within each SOA. In most of the negative SOA conditions, when flankers preceded a target, the lowest RT quantiles had the largest FCEs. This suggests that the conflict generated by early flankers interfered with target selection over a shorter time course, reducing the FCE$_{RT}$ in slower responses. The exception was the –400 ms condition; the flat slope of this delta plot suggests that flankers’ influence on target selection did not vary over time when presented 400 ms early. Despite the flat slope, the overall magnitude of the FCE$_{RT}$ was significantly different than zero, suggesting that even when flankers appear 400 ms before the target, they still impact target selection, but they do so consistently over time.

In conditions with targets preceding flankers, FCEs waned as SOA increased from 30 to 100 ms, as expected, and eventually disappeared when the flankers appeared 200 ms after the target. Our time course analysis showed that in most of these conditions, FCEs were greatest at the longest RTs (Fig. 3B). This overall result suggests that flanker processing occurred more slowly than target processing in these conditions. However, it is important to distinguish between the results produced in the +100ms and +200ms SOA conditions. Mean performance in both conditions indicated a lack of flanker interference (FCE = 0, Fig. 2), but our time course analysis revealed this effect was not consistent across the entire RT distribution in the +100ms SOA condition. Flankers did influence slow responses in the +100ms SOA condition but not in the +200ms SOA condition (Fig. 3B). The delta plot for +200ms SOA had a slope that did not differ significantly from zero, with FCE$_{RT}$ = 0 across the entire RT distribution. This suggests that the processing of task-relevant information, with no irrelevant information present, finishes within 200 ms because flankers shown 200 ms after target onset had no influence whatsoever. An interesting nuance here is that the decrease in mean FCE$_{RT}$ with increasing SOA was driven solely by faster responding on Incongruent trials, with little or no change in RTs observed on Congruent trials (Fig. 2B). This result suggests that congruent flankers do not facilitate or expedite selection, and that observed differences in the time course of selection are driven by interference dynamics.

## Experiments 2A & 2B

We designed our next experiments to yield additional, complementary perspectives on the time course of spatial selection. According to the zoom lens account, spatial selection occurs via a gradual narrowing of a Gaussian perceptual gradient (Eriksen & St. James, 1986; Hübner & Töbel, 2012; White et al., 2011). At trial onset in the flanker task, the selection gradient is centered over the target. As the selection gradient shrinks over the course of the trial, the perceptual weight given to each stimulus element varies dynamically: target weight increases and flanker weight decreases. Thus, the zoom lens account predicts that flankers have the most influence early on during the trial, when the extent selection gradient is widest. At later trial time points, the selection gradient has narrowed and flanker interference is inhibited. Our results from Experiment 1 were consistent with these predictions.

The zoom lens account further predicts that the selection process should dynamically interact with changes in the perceptual strength of flankers and targets. Increasing the strength of flanker input would have the largest effect early on in the trial, before the selection gradient has narrowed onto the target (Eriksen & St. James, 1986; Ulrich et al., 2015; White et al., 2011). To test this, we adjusted the contrast of targets and flankers at four different time points within each trial, compared to results from a control condition in which stimulus contrast was held constant at initial levels. In Experiment 2A, we increased flanker contrast; in Experiment 2B, that same increase in flanker contrast was accompanied by a concurrent reduction of target contrast. Both experiments tested how changes in the signal-to-noise (target-to-flanker) ratio would disrupt selection in real time.

**Fig. 4:**
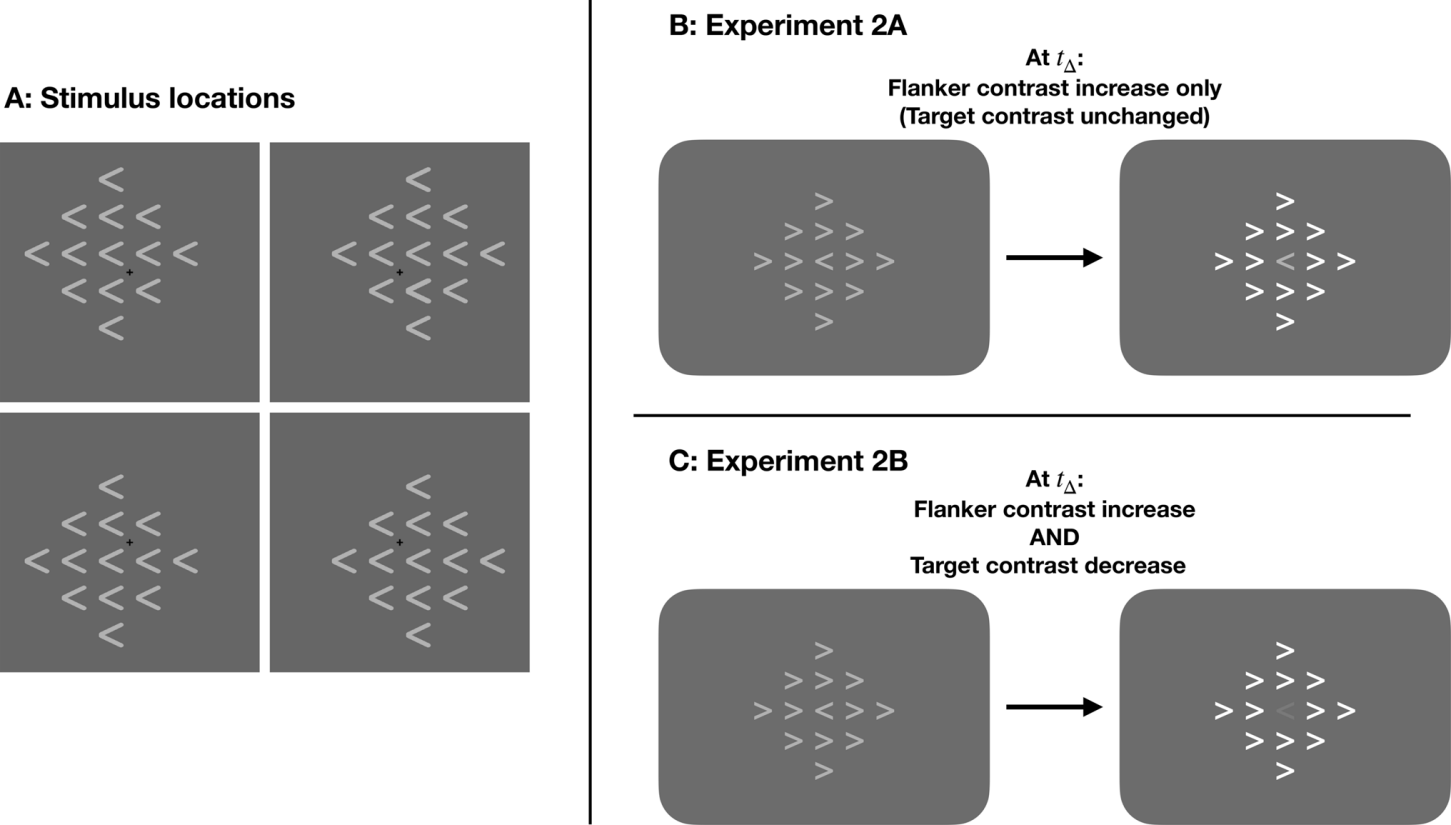
(**A**) Diagrams depicting the four possible stimulus locations and (**B**-**C**) the contrast manipulations used in the Dynamic trials of Experiments 2A & 2B, respectively). In both experiments, a change in overall stimulus contrast occurred at a trial time $t_\Delta$. In Experiment 2A, Dynamic trials included an increase in flanker contrast only, and target contrast was held constant at initial levels (**B**). In Experiment 2B, the contrast change involved both an increase in flanker contrast *and* a simultaneous decrease in target contrast (**C**). Brightness levels shown in the figure are for demonstration purposes only; see the main text for actual luminance values for each experiment and condition. Stimuli in these figures are not drawn to scale, and the grey backgrounds in each panel do not depict the full spatial extent of the display. See main text for details on stimulus and display size

By strengthening the flankers’ contrast (Experiment 2A, 2B) and weakening the target’s contrast (Experiment 2B), we rendered the perceptual weights set at trial onset less useful for accurate target selection, encouraging reactive selection adjustments. We therefore expected FCE magnitude to increase in all conditions with contrast changes, compared to the control condition. Specifically, because flanker interference is maximal early during stimulus display before selectivity has begun to develop (Ulrich et al., 2015; White et al., 2011), we hypothesized that introducing these contrast changes immediately after stimulus onset would produce the largest FCE. We expected contrast changes occurring later in the stimulus presentation to produce smaller FCEs, with the smallest FCE when a contrast change was absent altogether. We expected to observe these results in both experiments, with stronger effects in Experiment 2B, where the increase in flanker contrast was accompanied by reduced target contrast.

### Method

#### Subjects

Twenty-three subjects were recruited for each of Experiment 2A and Experiment 2B. We anticipated a moderately large effect size, similar to that reported by Kerns et al. (2022, $d = 0.93$). Our sample size gave us >95% power to detect such an effect. Subject demographics are shown in Table 1. One subject who had participated in Experiment 1 also participated in Experiment 2B. Subjects received course credit for participation.

#### Apparatus & stimulus

Subjects sat 60 cm from a 59.7-cm computer monitor (ASUS VH236H). The equipment, software, and stimulus configuration were identical to Experiments 1 and 3, except that stimulus elements were white rather than black. The choice of stimulus luminance is discussed in more detail below. Based on previous work by Kerns et al. (2022), to boost the size of the expected FCE, the location of the stimulus array varied among four possible locations. We used diagonal (rather than cardinal) displacements to counterbalance the lateralization of stimuli, as shown in Fig. 4A. To make sure that the fixation cross remained visible, the center of each stimulus array was presented ±.75° vertically and ±.75° horizontally, resulting in four possible stimulus locations centered 1.06° from fixation.

***Contrast manipulation*** To produce a wider range of stimulus-to-background contrasts, we used white arrowheads rather than the black arrowheads used in Experiments 1 and 3. All stimuli were presented on a gray background (46.5 cd/m^2^ luminance), with low (54.3 cd/m^2^), moderate (106.7 cd/m^2^), or high (209.6 cd/m^2^) contrast. Note the moderate level is the geometric mean of low and high levels.

In both experiments, elements in the stimulus array (the target arrowhead and the 12 surrounding flankers) were initially presented at the moderate contrast level as light gray arrowheads against a gray background (Fig. 4). The background remained unchanged throughout the experiments. In one condition (*Constant* contrast), all elements in the stimulus array remained at the initial, moderate contrast level for the entire stimulus duration. In another set of four conditions, which we refer to as *Dynamic* contrast conditions, either the flanker contrast (Experiment 2A) or both flanker and target contrast (Experiment 2B) changed. On Dynamic trials of both experiments, the flanker contrast increased. In Experiment 2A, target contrast remained fixed at the initial level on all trials (Fig. 4B). In Experiment 2B, the increase in flanker contrast was accompanied by a reduction in target contrast (Fig. 4C). We opted not to test a condition where target contrast was reduced with no change in flanker contrast because previous research found no significant effect of target strength alone on the FCE (Eriksen & Schultz, 1979; Servant et al., 2014).

In both experiments, the contrast change during Dynamic trials occurred at $t_\Delta =$ 33, 50, 67, or 83 ms after stimulus onset, selected to be during the window when selection processes are ongoing (Experiment 1). After the contrast change at $t = t_\Delta$, the stimulus elements remained at the new contrast levels for the remainder of the stimulus duration ($100 - t_\Delta$ ms). In the Constant condition, stimuli remained at the initial, moderate contrast level for the entire stimulus duration (i.e., $t_\Delta = none$), serving as a control condition in both experiments. The five possible $t_\Delta$ conditions (33 ms, 50 ms, 67 ms, 83 ms, or none) occurred with equal probability.

**Fig. 5:**
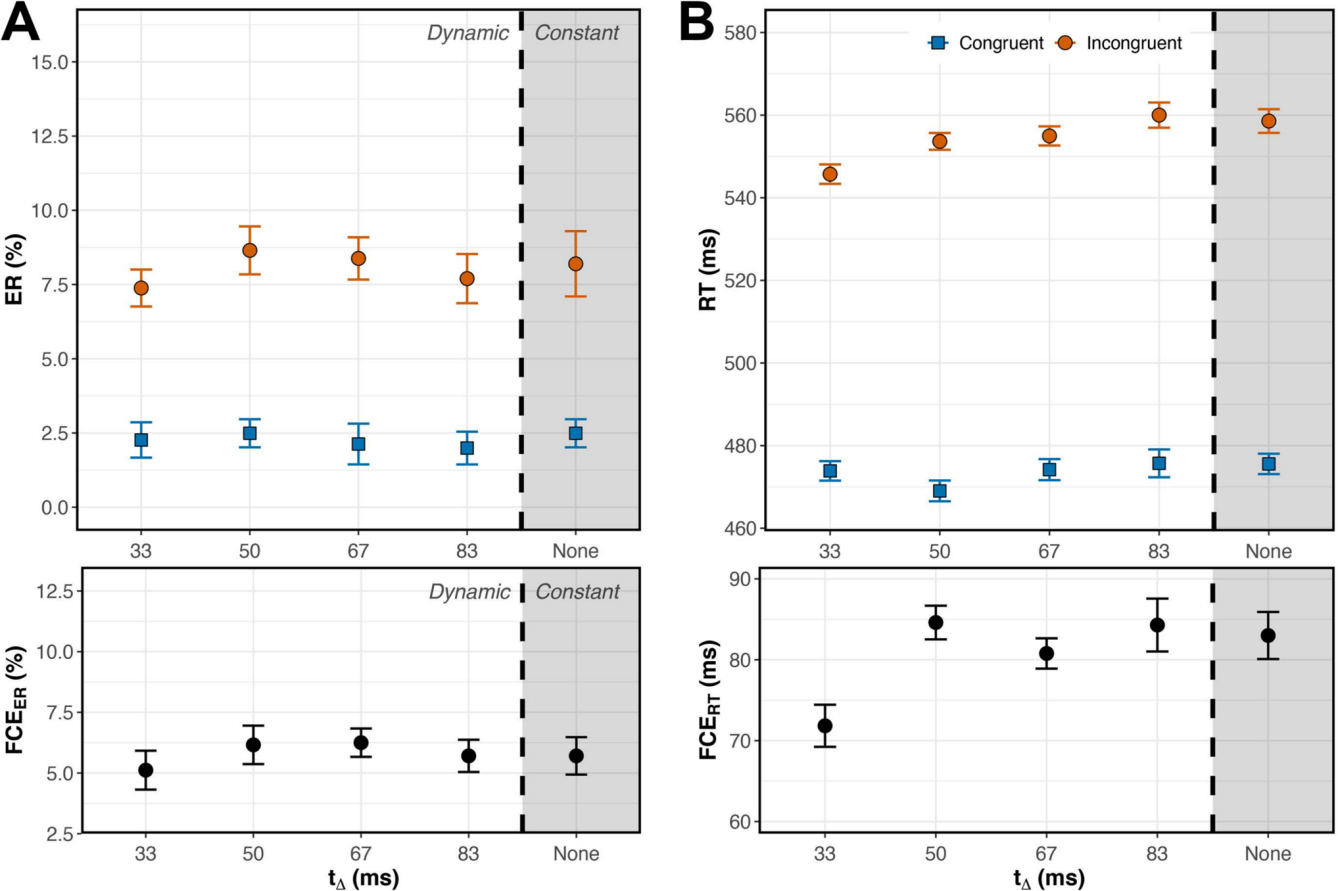
(**A**, top) Error rates were consistently lower on Congruent (blue, square) than Incongruent (orange, round) trials, but did not vary among $t_\Delta$ conditions. (**A**, bottom) The FCE$_{ER}$ did not vary in magnitude among $t_\Delta$ conditions. (**B**, top) RTs were shorter on Congruent than Incongruent trials and varied among $t_\Delta$ conditions. (**B**, bottom) RTs showed a significant Congruency $\times$ $t_\Delta$ interaction. Error bars reflect within-subject standard error, n=23

#### Task

After a fixation period that randomly varied between 1.05, 1.15, 1.25, and 1.35 s, the stimulus array was presented for 100 ms. As in the preceding experiments, subjects signaled their judgment of the target’s direction (leftward or rightward), and had up to 1 s to respond. After the response, feedback about response correctness was presented for 300 ms. Successive trials were separated by a mean inter-trial interval (ITI) of 1.5 s, which included feedback as well as the fixation period. The fixation cross remained on-screen throughout the trial and ITI.

Subjects completed 12 experimental blocks of 80 trials each. Within each block of trials, subjects saw each possible stimulus array (leftward- and rightward-directed targets crossed with Congruent or Incongruent flankers) presented four times at each $t_\Delta$ in random order. This yielded an experiment total of 96 trials per condition ($t_\Delta$ $\times$ Congruency) per subject. Before completing experimental blocks, subjects completed two practice blocks. The first practice block contained 16 Constant trials ($t_\Delta = none$). The second practice block contained 36 Dynamic trials (18 with $t_\Delta = 50 ms$ and 18 with $t_\Delta = 67 ms$). The Dynamic practice block did not include the most extreme $t_\Delta$ conditions.

Subjects received feedback after each trial and after each block, using the same procedures described in Experiment 1. We encouraged subjects to emphasize accuracy, maximizing any FCE$_{RT}$, by modifying the feedback score in Eq. 3 to weight accuracy at 80% and RT at 20%. As before, this composite score ranged from 0 to 100.

#### Data analysis

We used the same procedures to analyze mean error rate, response time, FCE$_{ER}$, FCE$_{RT}$, and the time course of the FCE as in Experiment 1.

### Experiment 2A Results & Discussion

In Experiment 2A (Fig. 5), we observed Congruency effects on error rate and RT, but $t_\Delta$ did not moderate these effects.

**Fig. 6:**
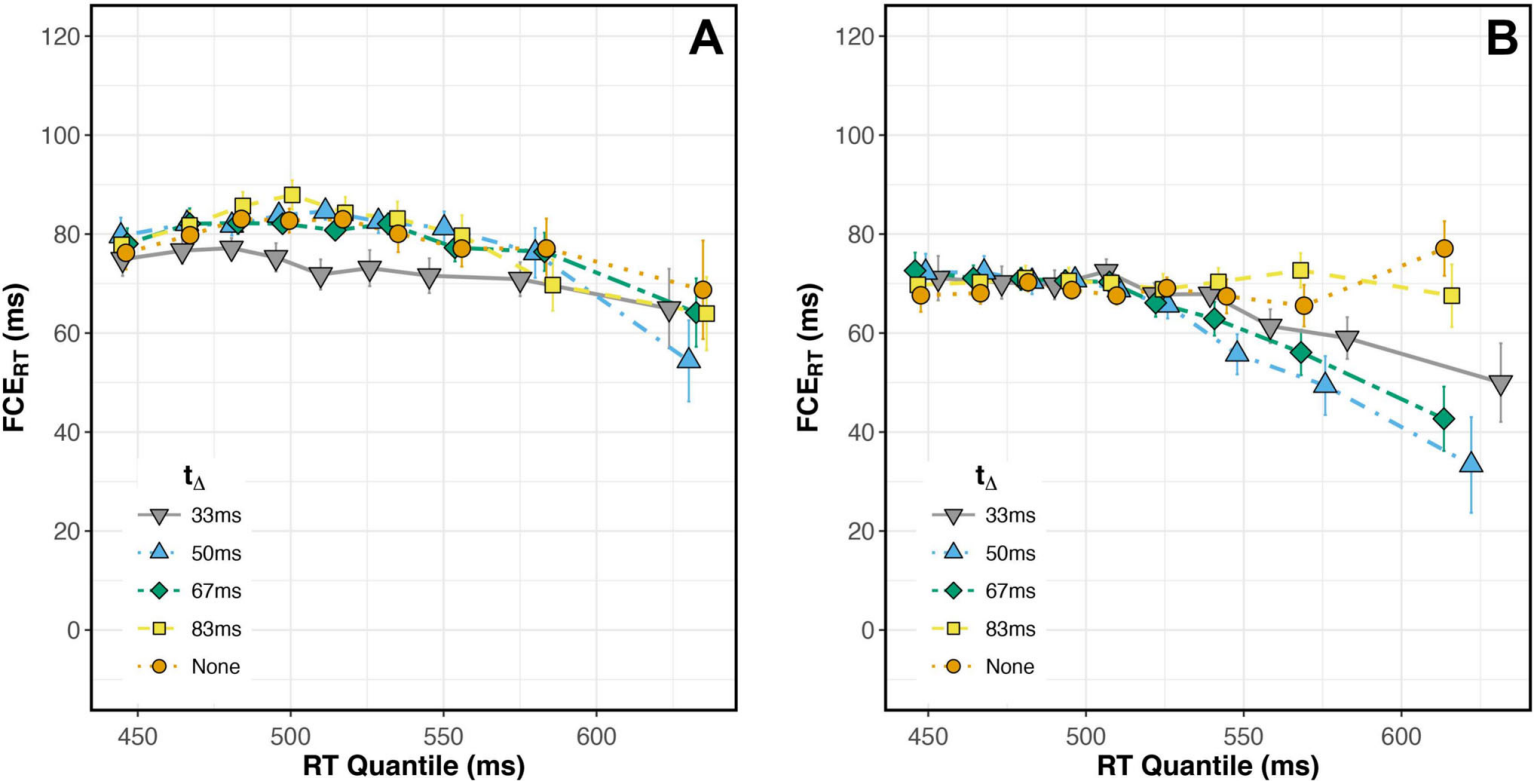
(**A**) Group average delta plots for the five $t_\Delta$ conditions in Experiment 2A, n=23. None of the delta plot slopes in Experiment 2A significantly differed from zero. (**B**) Group average delta plots for the five $t_\Delta$ conditions in Experiment 2B, n=23. Delta plot slopes were significantly negative when $t_\Delta$ = 50 ms (blue triangles) and $t_\Delta$ = 67 ms (green diamonds). All other delta plot slopes in Experiment 2B did not significantly differ from zero. Error bars reflect within-subject standard error

In the analysis of error rate, the main effect of Congruency was significant, $F(1,22) = 42.62$, $p <.001$, $\eta _p^2 =.66$ (Fig. 5A). Variations in $t_\Delta$ did not significantly affect mean error rate or FCE$_{ER}$ magnitude (main effect: $F(2.49,54.78) = 0.57$, $p =.608$, $\eta _p^2 =.03$; interaction: $F(3.29, 72.31) =.38$, $p =.782$, $\eta _p^2 =.02$). Analysis of RTs, however, yielded somewhat different results. On average, subjects had longer RTs on Incongruent trials, $F(1,22) = 503.74$, $p <.001$, $\eta _p^2 =.96$ (Fig. 5B). There was also a significant main effect of $t_\Delta$ on RTs, as well as a significant Congruency $\times$ $t_\Delta$ interaction (main effect: $F(2.49, 54.77) = 6.34$, $p =.002$, $\eta _p^2 =.22$; interaction: $F(3.16, 69.61) = 4.14$, $p =.008$, $\eta _p^2 =.16$). The significant interaction was driven by a reduced FCE$_{RT}$ when $t_\Delta$ = 33ms, compared to a larger FCE$_{RT}$ in other conditions (all $p <.05$). Delta plot slopes were not significantly different from zero in any condition (Fig. 6A).

We predicted that a mid-trial increase in flanker contrast would produce more interference and larger FCEs, with maximal effects for earlier contrast changes. However, we observed no effect of $t_\Delta$ on FCE$_{ER}$, and the direction of the $t_\Delta$ effect on FCE$_{RT}$ was the opposite of what we predicted. It is possible that perceptual grouping and/or segmentation played a part in producing these results (Banks & Prinzmetal, 1976; Treisman, 1982). Other studies have observed smaller FCEs with flankers that were perceptually distinct from targets along a task-irrelevant dimension (Moore et al., 2021), as is contrast in our experiment. In the present study, the contrast change at $t_\Delta = 33$ ms could have occurred early enough to enhance image segmentation, facilitating spatial selection and faster binary decisions about the target’s direction (Moore et al., 2021). When flanker contrast increased later in the stimulus presentation (or not at all, in the Constant condition), spatial selection may not have benefited, producing larger FCE$_{RT}$ in all other conditions.

### Experiment 2B: Results & Discussion

**Fig. 7:**
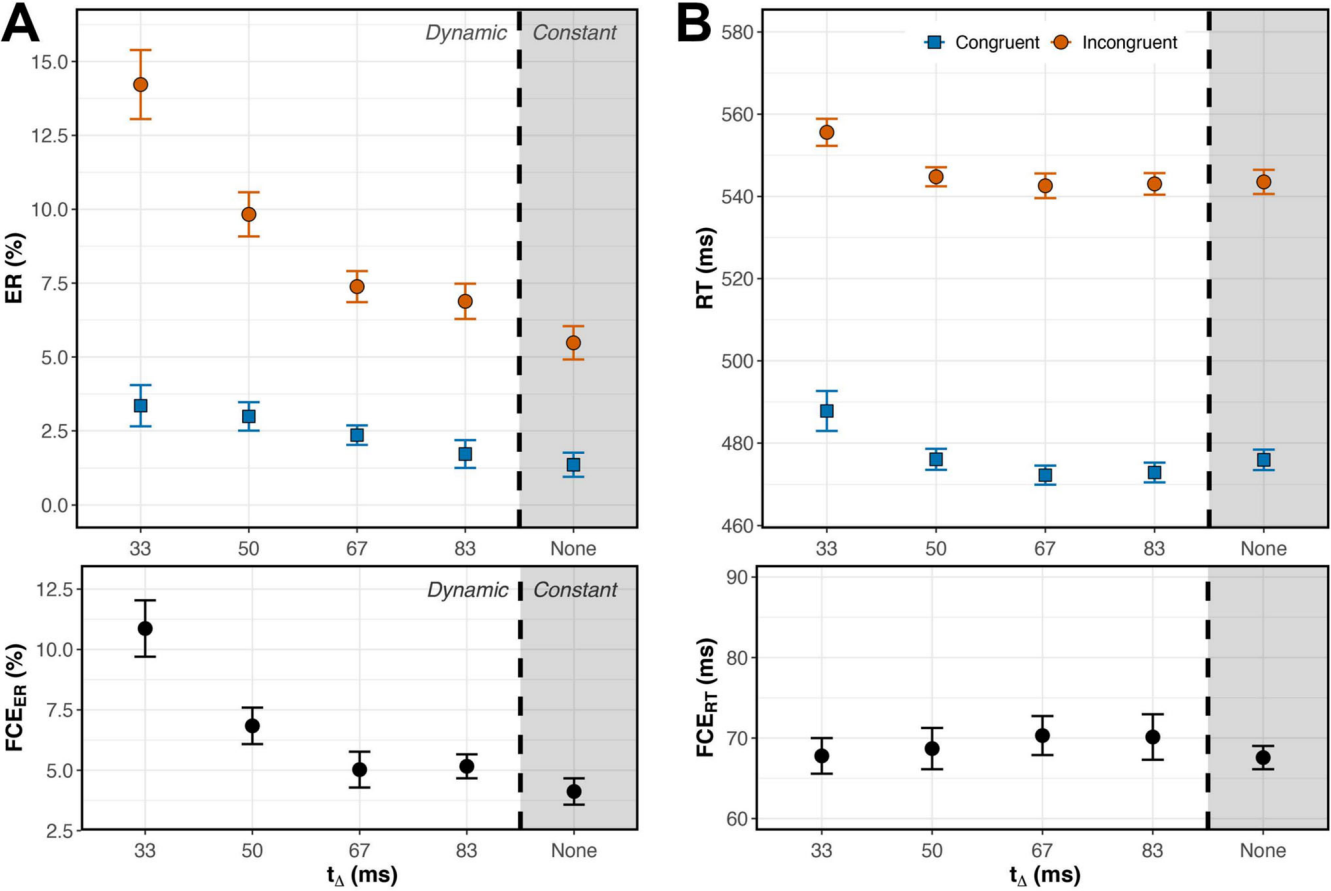
(**A**, top) Error rates were consistently lower on Congruent (blue, square) than Incongruent (orange, round) trials and varied among $t_\Delta$ conditions. (**A**, bottom) Mean FCE$_{ER}$ varied in magnitude among $_\Delta$ conditions, with earlier $_\Delta$ producing larger FCE$_{ER}$. (**B**, top) RTs were shorter on Congruent than Incongruent trials and varied among $_\Delta$ conditions. (**B**, bottom) There was not a significant Congruency $\times$ $t_\Delta$ interaction for FCE$_{RT}$s. Error bars reflect within-subject standard error, n=23

Experiment 2B’s concurrent changes in both target and flanker contrast led to a different pattern of results than in Experiment 2A. Here, $t_\Delta$ affected FCE$_{ER}$ magnitude as we hypothesized (Fig. 7A). Subjects made significantly more errors on Incongruent trials, regardless of $t_\Delta$, $F(1,22) = 134.44$, $p <.001$, $\eta _p^2 =.86$. Mean ER also significantly differed across $t_\Delta$ conditions, $F(2.26,49.74) = 20.89$, $p <.001$, $\eta _p^2 =.49$. Importantly, the magnitude of FCE$_{ER}$ depended on $t_\Delta$, $F(2.27, 49.88) = 11.88$, $p <.001$, $\eta _p^2 =.35$. We observed the largest FCE$_{ER}$ on trials with the earliest contrast change; then, as the contrast change was delayed, FCE$_{ER}$ decreased, reaching a minimum on trials with no contrast change.

Subjects in Experiment 2B tended to respond more slowly on Incongruent trials $F(1,22) = 333.73$, $p <.001$, $\eta _p^2 =.94$ (Fig. 7B). There was also a significant main effect of $t_\Delta$ on RTs, $F(1.53, 33.70) = 8.46$, $p =.002$, $\eta _p^2 =.28$. We did not observe a significant Congruency $\times$ $t_\Delta$ interaction, $F(3.10,68.31) =.30$, $p =.833$, $\eta _p^2 =.01$. FCE$_{RT}$ magnitude was similar across all $t_\Delta$ conditions. Our time course analysis revealed, however, that earlier contrast changes (lower values of $t_\Delta$) actually led to *reduced* FCE$_{RT}$ for the slowest responses (Fig. 6B). Delta plot slopes were significantly negative when $t_\Delta = 50$ ms and $t_\Delta = 67$ ms ($t(22) = -2.77$, $p =.028$, $d = -.58$; and $t(22) = -3.49$, $p =.010$, $d = -.73$; respectively). Delta plot slopes were not significantly different from zero in the other three conditions.

The only difference between Experiments 2A and 2B was whether target contrast remain at initial levels throughout the trial or instead decreased at $t_\Delta$. This single change produced markedly different results: in Experiment 2B, $t_\Delta$ significantly affected FCE$_{ER}$ magnitude in agreement with our prediction that earlier $t_\Delta$ would produce larger FCEs (Fig. 7A). We did not see the same effect on mean FCE$_{RT}$ magnitude (Fig. 7B), but delta plots revealed that earlier contrast changes led to *reduced* FCE$_{RT}$s for the slowest responses (Fig. 6B). These two results support contradictory hypotheses. Larger FCE$_{ER}$ with earlier contrast changes suggest that our contrast manipulations reduced the signal-to-noise ratio and *increased* conflict, as we initially hypothesized. *Smaller* FCE$_{RT}$s on trials with earlier contrast changes, as we observed through time course analysis, suggest that the contrast manipulation may have instead *reduced* conflict by facilitating image segmentation (Moore et al., 2021) and/or perceptual grouping (Banks & Prinzmetal, 1976; Treisman, 1982). These inconsistent results have complex implications for how conclusions drawn from ER and RT differ and how selection can be affected by stimulus modifications after onset, suggesting a potential avenue for future research.

## Experiment 3

Experiments 1 and 2 showed that temporal manipulations had a substantial effect on visual selection; Experiment 3 followed up by examining the effects of a spatial manipulation, stimulus location uncertainty. Proactive attention to the location at which a stimulus will appear enhances both detection and feature processing (Downing, 1988; Leber et al., 2016; Serences et al., 2005), and sensitivity falls off rapidly with distance from the attended focus. Further, shifting the focus of attention takes a non-zero amount of time (Posner et al., 1980). Thus, increasing subjects’ uncertainty about the location of an upcoming stimulus arrangement should increase the FCE, as subjects must rely more heavily on reactive control mechanisms to guide attention to the target. Kerns et al. (2022) tested this for large stimulus movements using an online study and observed the predicted increase in FCE. Here, we wanted to replicate their finding under laboratory conditions that afforded more control over the amount of spatial uncertainty. We also tested whether guiding spatial attention to a random location is a functionally distinct stage of processing that occurs in sequence with target selection (Sternberg, 2011).

In Experiment 3, we manipulated whether the stimulus appeared at the same location from trial-to-trial or instead appeared randomly at one of four possible locations. We used a relatively modest amount (< 1°) of spatial uncertainty to test the limits of Kerns et al. (2022)’s observed effect. In addition to comparing FCE magnitude between conditions with and without location uncertainty, we also investigated whether there were supra-trial effects of location among uncertain trials (akin to a Gratton effect; Gratton et al., 1992). Exploiting the expected effect of spatial uncertainty, we tested whether having the stimulus appear at the same location of successive trials affected the efficacy of spatial selection. We found that varying stimulus location unpredictably from trial to trial negatively impacted selection, resulting in increased average response time, error rate, and FCE magnitude. We also found evidence in support of the conclusion that guiding spatial attention and target selection are not functionally distinct stages of processing.

### Method

#### Subjects

We collected data from 22 Brandeis University undergraduates (Table 1). We expected to see an effect size similar to that reported by $d = 0.93$; Kerns et al. (2022). Our sample size gave us >95% power to observe the same effect. Subjects received course credit for participation.

**Fig. 8:**
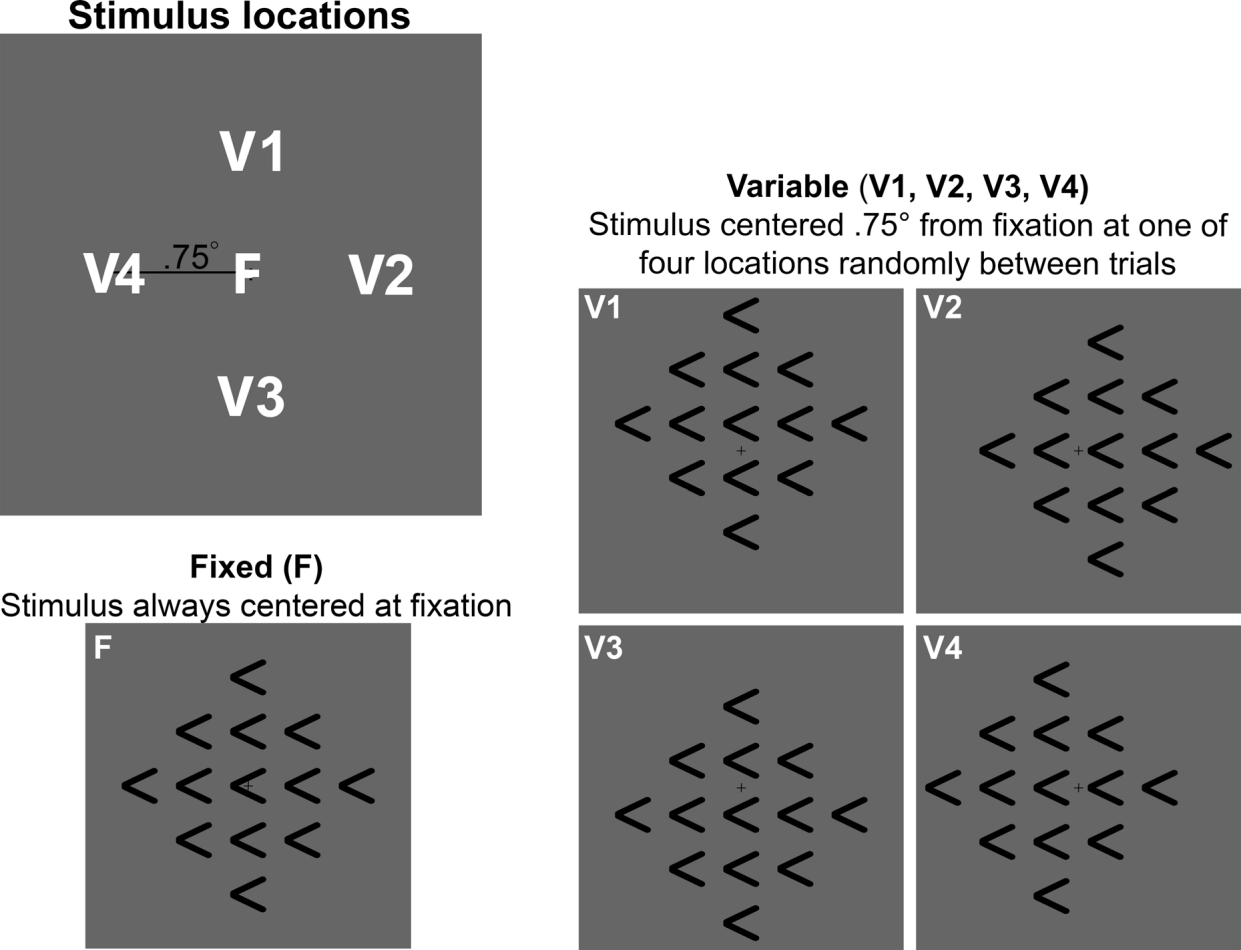
Diagrams of the possible stimulus locations in Experiment 3 (not drawn to scale). The white letters and numbers in each panel are for illustrative purposes only; subjects saw only the stimulus elements and the fixation cross. In the Fixed (F) condition, the stimulus array was presented centered at fixation on every trial. In the Variable conditions, stimulus location varied randomly from trial to trial and could take any of four possible locations (V1-V4). For each Variable location, the entire stimulus array shifted, causing the target’s location in the array’s center to be displaced by.75 ° from fixation in one of four cardinal directions (above, below, right, or left). Note that the grey backgrounds in each panel do not depict the full spatial extent of the display. See main text for details on display size

#### Apparatus & stimulus

We used the same apparatus configuration as in Experiment 2. As in our previous experiments, the stimuli were arrays of 13 arrowheads (one central target surrounded by 12 flankers), each subtending 1° visual angle. Individual arrowheads were separated by 1.5° center-to-center. Distance between arrowheads was increased compared to our previous experiments to ensure the fixation cross remained completely visible in conditions with spatial variability of the stimulus. Mean arrowhead luminance was 0.7 cd/m^2^, presented against a middle-gray background (29.6 cd/m^2^). The target arrowhead pointed either left () or right (), and was surrounded by flankers whose directions were either all Congruent or Incongruent with that of the target.

Our primary manipulation was stimulus location uncertainty (Fig. 8). In Fixed blocks, the target and flankers were presented at the same location on every trial, with the target position always set centrally at fixation. In Variable blocks, the position of the entire stimulus array (target and surrounding flankers) varied randomly among four possible locations, each.75° visual angle away from fixation along one of the cardinal directions (Fig. 8). This variability allowed us to characterize uncertainty at two timescales: globally (block level) and locally (trial level). While there was global uncertainty throughout a Variable block, some trials’ location was identical to that on the trial immediately prior (Variable-Same trials), leading to locally reduced uncertainty. Other trials’ location differed from that on the immediately preceding trial (Variable-Diff trials), leading to locally increased uncertainty.

To encourage subjects not to make eye movements on Variable trials, a fixation cross remained on the screen at all times. The brief, 100 ms stimulus presentation made it unlikely that a subject could initiate and complete a saccade while the stimulus was visible. To reduce the likelihood of a subject mistaking a flanker for the target on Variable trials, we used a small,.75° spatial shift that prevented a flanker arrowhead from falling on the fixation cross, as shown in Fig. 8.

#### Task

Trials were presented in 12 blocks. Each block contained 80 trials, with all stimuli in a block presented either (i) at fixation (*Fixed* blocks) or (ii) at one of four offset locations that was selected on each trial (*Variable* blocks). Each trial began with the target and flankers appearing simultaneously, and both remained on the screen for 250 ms. Subjects had up to 1.25 s from stimulus onset to respond. We used the same stimulus–response mappings as in previous experiments. After each response, visual feedback was given for 300 ms, followed by a 1.1$-$1.4 s fixation period (mean duration = 1.25 s). Subjects again received a feedback score between 0 and 100 after each block. We encouraged subjects to emphasize accuracy, maximizing any FCE$_{RT}$, by modifying the feedback score in Eq. 3 to weight accuracy at 75% and RT at 25%.

Blocks alternated between Fixed and Variable conditions. Subjects completed three repetitions of either an ABBA or BAAB reverse counterbalanced order, yielding 480 trials per subject in each Location condition.

#### Data analysis

To analyze trial-by-trial carry-over effects occurring at the local timescale, the first trial from each block was excluded. This yielded 474 Fixed Location trials and 474 Variable Location trials per subject. Variable trials were further divided into Variable-Same and Variable-Diff trial groups. Because stimulus position was pseudo-randomly selected from the four alternatives on each Variable trial, the number of Variable-Same and Variable-Diff trials varied across subjects. On average, subjects completed a total of 114 Variable-Same trials (SD: 10.67, range: 100-138 trials) and 359 Variable-Diff trials (SD: 10.67, range: 336-374 trials). We analyzed ERs, RTs, and FCEs using the same procedures as in our previous experiments.

**Fig. 9:**
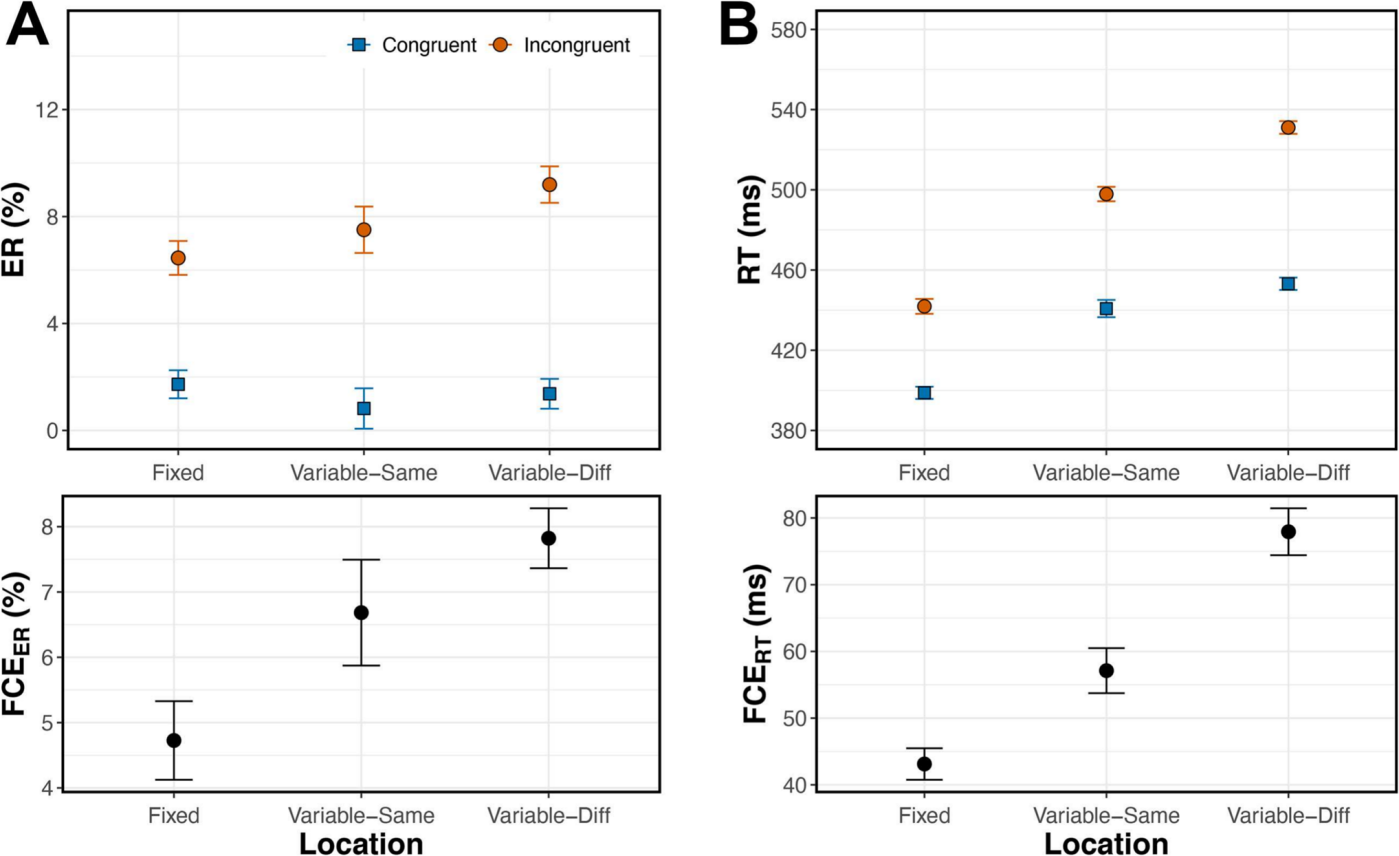
Within Fixed blocks in Experiment 3, the stimulus was presented at fixation on every trial. Within Variable blocks, stimulus location was either identical to that of the trial immediately prior (Variable-Same) or differed from that on the trial immediately prior (Variable-Diff). (**A**, top) Error rates were lower on Congruent than Incongruent trials and varied among Location conditions. (**A**, bottom) There was also a significant Congruency $\times$ Location interaction, and FCE$_{ER}$ was largest on Variable-Diff trials, when there was both global and local uncertainty. (**B**, top) Response times were shorter on Congruent than Incongruent trials and varied among Location conditions. (**B**, bottom) There was also a significant Congruency $\times$ Location interaction; FCE$_{RT}$ was largest on Variable-Diff trials, when there was both global and local uncertainty. Error bars reflect within-subject standard error, n=22

### Results

Figure 9 shows that spatial uncertainty significantly affected error rate, response time, and both measures of the FCE. Omnibus ANOVA results for both dependent measures are reported in Supplementary Table 3.

#### Error rate

Mean ER differed among the three Location conditions (Fig. 9A, Supplementary Table 3). There was also a significant main effect of Congruency, as well as a significant Location $\times$ Congruency interaction, suggesting that FCE$_{ER}$ differed among Location conditions. *Post hoc* comparisons confirmed that there was a significant FCE$_{ER}$ in each condition (Fixed: 4.7%, $p <.001$, $\eta _p^2 =.56$; Variable-Same: 6.7%, $p <.001$, $\eta _p^2 =.54$; Variable-Diff: 7.8%, $p <.001$, $\eta _p^2 =.71$; Fig. 9A).

Focusing on differences between blocks (Fixed vs. Variable), the Variable-Diff FCE$_{ER}$ was significantly larger than the Fixed FCE$_{ER}$, one-tailed $p <.001$, $\eta _p^2 =.58$. The FCE$_{ER}$ in the Variable-Same condition did not significantly differ from the Fixed condition, $p =.130$, $\eta _p^2 =.13$. *Post hoc* comparison showed that the mean Variable FCE$_{ER}$ significantly differed from the mean Fixed FCE$_{ER}$ overall, $F(1, 16) = 11.67$, $p =.004$, $\eta _p^2 =.42$. Within Variable blocks, FCE$_{ER}$ did not significantly differ between Variable-Same and Variable-Diff conditions, $p =.247$, $\eta _p^2 =.06$. *Post hoc* comparisons showed that Congruent ER did not differ among Location conditions (all $p >.05$), the changes in FCE$_{ER}$ magnitude were driven by the different ERs on Incongruent trials, namely between Fixed and Variable-Diff conditions (2.7% mean difference, $p <.001$, $\eta _p^2 =.51$).

**Fig. 10:**
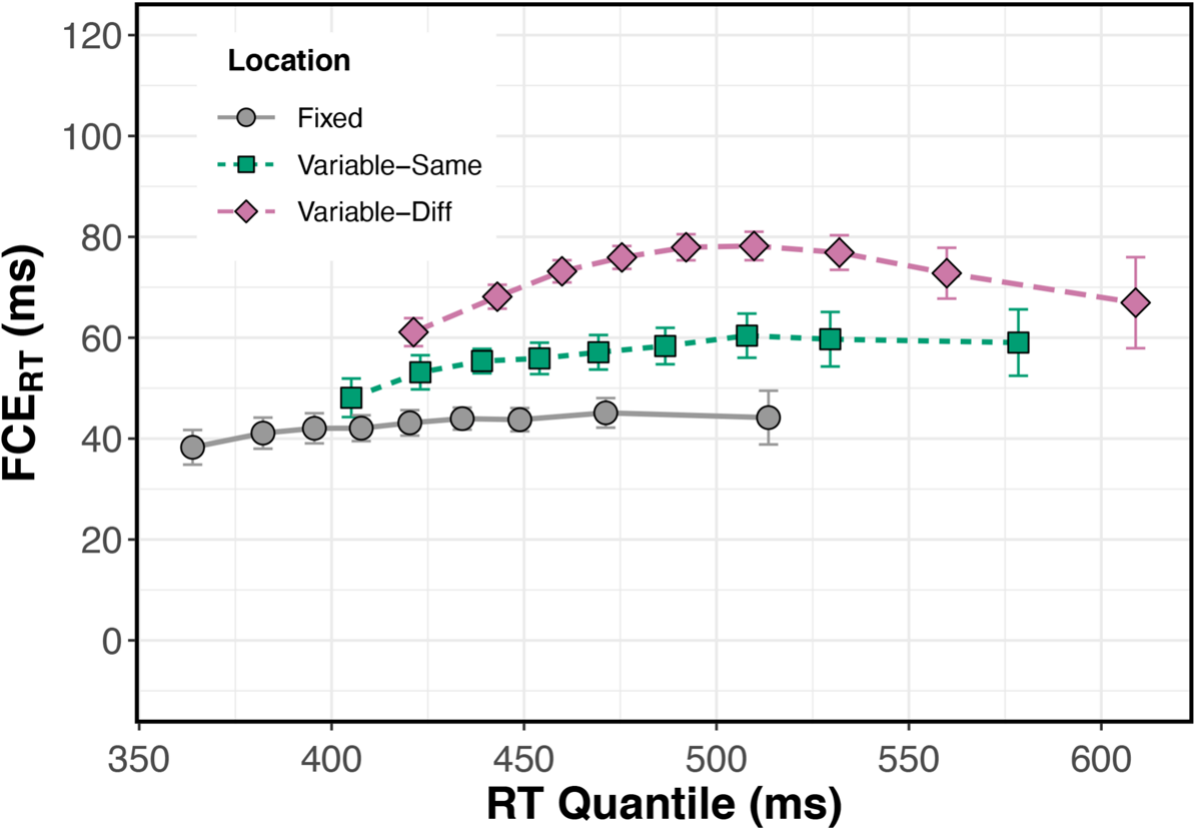
Delta plots for the three Location conditions in Experiment 3. Within Fixed blocks, the stimulus was presented at fixation on every trial. Within Variable blocks, stimulus location was either identical to that of the trial immediately prior (Variable-Same) or differed from that on the trial immediately prior (Variable-Diff). Error bars reflect within-subject standard error, n=22

#### Response time

The mean RT differed among the three Location conditions and between Congruent and Incongruent conditions (Fig. 9B, Supplementary Table 3). There was also a significant Location $\times$ Congruency interaction, suggesting that FCE$_{RT}$ differed among Location conditions. Post hoc comparisons showed a significant FCE$_{RT}$ in all Location conditions (Fixed: 43 ms, $p <.001$, $\eta _p^2 =.88$; Variable-Same: 57 ms, $p <.001$, $\eta _p^2 =.81$; Variable-Diff: 78 ms, $p <.001$, $\eta _p^2 =.92$; Fig. 9B).

We found evidence of significant modulations of FCE$_{RT}$ occurring on the global timescale. FCE$_{RT}$ in Variable-Same and Variable-Diff conditions were significantly larger than in the Fixed condition, one-tailed $p =.001$, $\eta _p^2 =.39$ and $p <.001$, $\eta _p^2 =.78$, respectively. There was also significant evidence of local, within-block effects: FCE$_{RT}$ was significantly larger in the Variable-Diff condition than in the Variable-Same condition, one-tailed $p =.001$, $\eta _p^2 =.42$.

We additionally compared the carry-over effect (i.e., the difference between Variable-Same and Variable-Diff RTs) on Congruent trials to the carry-over effect on Incongruent trials. Carry-over effect magnitude significantly differed between Congruency conditions by 21 ms, 95% CI: (10 ms, 32 ms), $t(21) = 3.94$, $p <.001$, $\eta _p^2 =.42$.

#### Time course of FCE

Delta plots showing the time course of spatial selection for the three Location conditions are shown in Fig. 10. Delta plot slope did not significantly differ from zero in any condition (all $p >.05$); however, visual inspection of the plots showed variation in shape across the conditions. We subsequently ran *post hoc* polynomial contrasts for each condition to test for linear and quadratic trends. Only the linear trend was significant for the Fixed ($t(174) = 2.2$, $p =.028$) and Variable-Same ($t(174) = 2.7$, $p =.007$) conditions. In contrast, only the quadratic trend was significant for the Variable-Diff condition ($t(174) = -4.4$, $p <.001$).

### Discussion

Overall, subjects made more errors and responded more slowly when uncertain where the stimulus would appear. We also found that spatial uncertainty increased FCE magnitude in both RTs and ERs, even with only a modest shift of stimulus location (.75 ° from fixation). Global-scale uncertainty about location evoked varying degrees of reactive modulation of spatial selection, evidenced by the difference in FCE magnitude between Variable-Same and Variable-Diff conditions.

Comparing RTs between Variable-Same and Variable-Diff trials in this way afforded a more nuanced view of how spatial uncertainty affects target selection in the flanker task. In both conditions, subjects did not know where the stimulus would appear. Still, they responded faster and made fewer errors when the stimulus appeared in the exact location as in the previous trial, demonstrating *reactive*, within-trial modulations of spatial selection. These reactive modulations of spatial selection occurred concurrently with *proactive* block-to-block effects demonstrated by the difference between Fixed and Variable FCE magnitude. That FCE$_{RT}$ magnitude was smaller on Variable-Same compared to Variable-Diff trials suggests that spatial attention is either deployed more effectively or remains deployed at a recently attended location between trials, despite all spatial locations having equal likelihood. We discuss the implications of these findings further in the General Discussion.

## General discussion

Our four variants of the Eriksen flanker task traced the earliest phases of visual-spatial selection’s dynamics. Our principal results demonstrated that the flanker congruency effect (FCE) depends upon transient intra-trial factors, including (i) the details of flanker and target timing and (ii) modulation of flanker and target contrast. Our results also revealed the impact of longer-term proactive factors, like (iii) uncertainty about stimulus location from trial to trial, both at the local level of trial sequence (trial-to-trial carryover) as well as within a global context (differences between blocks). Together, these results suggest that spatial selection processes are vulnerable to multiple sources of interference and that this vulnerability fluctuates over time, both within- and between-trials. The following discussion explores how our findings support a mechanistic understanding not only of the flanker task but also of spatial selective attention more generally.

### Within-trial adjustments of selection

In the flanker task, spatial selection is usually indexed by the mean FCE. That summary statistic reflects performance aggregated over many trials and is, therefore, mute about what happens during an individual trial. To investigate within-trial dynamics using behavioral data, we drew heavily upon the detailed information embedded in response time distributions.

Examining the FCE$_{RT}$ across RT distributions, we found a subset of conditions that produced negatively sloped delta plots. In those conditions, flanker interference was largest for fast responses and shrank when subjects took more time to respond. Two hypotheses have been offered to account for reduced congruency effects in slower responses. According to the activation-suppression hypothesis (Ridderinkhof, 2002), negatively-sloped delta plots reflect active suppression of interfering information, with steeper slopes tracking the strength of the suppression. Alternatively, reduced congruency effects in slower responses may be brought about by the passive decay of flanker information (Ulrich et al., 2015).

Evaluating Experiment 2B’s delta plots against each of these hypotheses, the data seem to favor an active suppression mechanism over passive decay. The flat delta plot in the Constant condition of Experiment 2B suggests that neither suppression nor decay was recruited when the stimulus remained at initial contrast levels for the entire trial. In the Dynamic conditions of Experiment 2B, only contrast shifts occurring within 67 ms of stimulus onset produced negative-going delta plots (Fig. 6B). The contrast shift strengthened flanker influence while also degrading target input, with the intention of disrupting spatial selection. It is unlikely that increasing flanker contrast would somehow lead to faster passive decay. More plausibly, slower responding allowed an active suppression mechanism to successfully reduce the impact of higher contrast flankers.

In order to better understand selection mechanisms in this task, we compared the intercept of each negatively-sloped delta plot to that of the control condition in each experiment (SOA = 0 ms in Experiment 1; $t_\Delta$ = 0 in Experiment 2B). Looking at fast responses in Experiment 1 (short RTs; left-most points in Fig. 3A), early flanker onset (relative to target onset) impeded selection processes compared to the control condition, as shown by the larger FCEs when SOA< 0 ms. When subjects responded more slowly (long RTs; right-most points in Fig. 3A), selection occurred as effectively with early flanker onset as with simultaneous flanker and target onset. Interestingly, our manipulation in Experiment 2B encouraged a suppression mechanism that led to *more effective* selection compared to the control condition, producing *smaller* FCEs in slower responses (right-most points in Fig. 6B). So, even though delta plots from both studies were negatively-sloped, comparing their intercepts relative to control conditions suggests differences in within-trial selection dynamics between the experiments.

### Between-trial adjustments of selection

Experiments 1 & 2 focused on reactive effects, spatial selection that occurs within a trial. Experiment 3 demonstrated proactive selection adjustments in response to broader context. Specifically, we found that context mattered not only *between* Fixed and Variable blocks of trials, but also in pairs of trials *within* Variable blocks. This carry-over effect highlights the role of recent experience in spatial attention, suggesting a form of spatial inertia in the selection process (Dube & Golomb, 2021; Narhi-Martinez et al., 2023; Nobre & Stokes, 2019). That is, target selection on one trial increases selection efficiency on the following trial if the next target appears at that same location. One possible way to explain such a carry-over effect between trials in the context of the zoom lens framework is that, in conditions with spatial uncertainty, engaging with the stimulus on one trial narrows the attentional field enough to serve as a pre-cue for the next trial (Anderson et al., 2021). Alternatively, carry-over effects may reflect top-down attentional control mechanisms that promote the processing of internally activated templates. This account treats the effect as resulting from a form of response bias (Beck & Kastner, 2009). Future efforts to expand and refine the zoom lens account should include selection history and bias effects, particularly when considering reward-modulated fluctuations of selection, due to the strength of reward in attentional modulation (Anderson et al., 2021; Failing & Theeuwes, 2018; Frömer & Shenhav, 2022; Hassett & Hampton, 2022).

Carry-over effects like those seen in Experiment 3 can help resolve key details of how spatial selection unfolds over time mechanistically. When stimulus location is uncertain, observers must both *shift* their focus of attention as well as *narrow* it onto the target. These processes have previously been considered to occur distinctly during selection (Bartsch et al., 2023; Treue & Martinez-Trujillo, 2012), but it is not known whether they must occur serially. It may be that stimulus onset evokes a shift of spatial attention so rapid that it is essentially complete before detailed stimulus processing (i.e., narrowing) begins. Alternatively, spatial attention may be broadly distributed at trial onset, such that any shift or re-centering occurs in parallel to information extraction, during the trial. We observed Congruency-specific carry-over effects in Experiment 3 where the local effect of uncertainty had a larger effect on selection under Incongruent than Congruent conditions. This finding suggests that the extraction of Congruency information and Location information overlapped, supporting the hypothesis that spatial selection operates *in parallel with* the extraction of perceptual information during the trial, rather than as strictly serial operations.

### Bridging complementary perspectives

Although we have structured our investigation of spatial selection contrasting reactive and proactive timescales, our results also suggest that this binary distinction is too simplistic. For example, although between-trial effects can be set in motion before a trial, as in Experiment 3, that does not preclude further modification during a trial, as in Experiments 1 and 2. We began to demonstrate this synthesis across timescales in Experiment 3, where we showed between-block effects were further modulated within-block.

Our results highlight the overall significance of the temporal domain in investigations of selection mechanisms. Temporally predictable structures like rhythms and repeated sequences comprise regularities that aid selective attention (see Nobre & van Ede, 2018, for a review). These same temporal structures could be systematically perturbed to better understand their significance in spatial selection and extraction of visual information. For example, Experiment 1 results suggest that irregularly timed flanker stimuli impact target selection. Our findings, combined with temporal rhythms’ ability to impact attention and expectation, suggest that temporal uncertainty (in addition to *spatial* uncertainty) might critically impact the effectiveness of selection mechanisms. Future work would benefit from investigating whether the observed effects replicate under varying degrees of temporal regularity.

### Conclusion

The present study adds to the multiple lines of evidence converging on the idea that attentional selection has a complex topography (Cave & Bichot, 1999; Datta & DeYoe, 2009; Müller & Hübner, 2002; Weichart & Sederberg, 2021). We designed this investigation to foster a mechanistic, theory-guided dissection of spatial selection. When considered within the body of spatial attention research, the present study offers several theoretically significant stimulus factors that affect what Narhi-Martinez et al. (2023) refer to as the “multi-level system of weights and balances” of attention. Our findings demonstrate that spatial selection in the flanker task is dynamically modulated, with significant effects produced at a wide range of timescales. These results highlight the need for future investigations of spatial selection to move beyond the simplistic dichotomy of between- and within-trial effects.

## Supplementary Information

Below is the link to the electronic supplementary material.

Supplementary file 1 (docx 23 KB)

## Data Availability

The trial data and subject-level data for all experiments are available at https://osf.io/72q9d/.

## Declarations

### Open Practices Statement

The trial data, subject-level data, and analysis code for all experiments are available at https://osf.io/72q9d/.
