# Supplementary material for: Dynamic modulation of spatial selection: Online and anticipatory adjustments in the flanker task
Source: Atten Percept Psychophys. 2025 Feb 20;87(3):794–814. doi: 10.3758/s13414-025-03026-5 (PMC11965244; doi:10.3758/s13414-025-03026-5)
Supplement: Supplementary file 1 — (docx 23 KB) [file 13414_2025_3026_MOESM1_ESM.docx]

**﻿Dynamic modulation of spatial selection: Online and anticipatory adjustments in the flanker task**

**﻿**Mercedes B. Villalonga, Abigail L. Noyce, Robert Sekuler

**Supplementary Tables 1-3**

**Supplementary Table 1**

***Mixed Design ANOVA Results for Experiment 1: Effects of Group × Congruency × SOA***

|  | Group Mean Error Rate (95% CI), % | | | | Group Mean - Response Time (95% CI), ms | | | |
| --- | --- | --- | --- | --- | --- | --- | --- | --- |
|  | Paid (n=15) | | Course Credit (n=25) | | Paid | | Course Credit | |
| Congruent | 1.93 (1.13, 2.72) | | 1.93 (1.31, 2.54) | | 372 (357, 386) | | 370 (359, 381) | |
| Incongruent | 9.06 (7.12, 11.0) | | 7.95 (6.45, 9.46) | | 417 (399, 434) | | 409 (395, 422) | |
| Effect | Error Rate | | | | Response Time | | | |
|  | *df* | *F* | *p* | η_p_^2^ | d*f* | *F* | *p* | η_p_^2^ |
| Group | 1, 38 | .51 | .480 | .01 | 1, 38 | .25 | .617 | .01 |
| Congruency | 1, 38 | 167.60 | **< .001** | .82 | 1, 38 | 450.56 | **< .001** | .92 |
| SOA | 3.3, 124.3 | 28.98 | **< .001** | .43 | 3.6, 135.6 | 75.62 | **< .001** | .67 |
| Group × Congruency | 1, 38 | 1.18 | .285 | .03 | 1, 38 | 2.47 | .124 | .06 |
| Group × SOA | 3.3, 124.3 | .76 | .527 | .02 | 3.6, 135.6 | 2.23 | .076 | .06 |
| SOA × Congruency | 3.0, 115.3 | 27.71 | **< .001** | .42 | 4.8, 180.8 | 53.11 | **< .001** | .58 |
| Group × SOA × Congruency | 3.0, 115.3 | 1.50 | .219 | .04 | 4.8, 180.8 | 1.84 | .111 | .05 |

Note:

SOA, Group × SOA, SOA × Congruency, and Group × SOA × Congruency effects corrected for sphericity using Greenhouse-Geisser method.

**Supplementary Table 2**

***Post Hoc Comparisons Among Stimulus Onset Asynchrony (SOA) Conditions in Experiment 1***

| SOA Comparison | FCE_ER_, Pr(correct) | | | FCE_RT_, milliseconds | | |
| --- | --- | --- | --- | --- | --- | --- |
|  | Mean diff. (95% CI) | *t*(39) | *p* | Mean diff. (95% CI) | *t*(39) | *p* |
| -50ms vs -100ms | .015 (-.031, .061) | 1.03 | .309 | 7.5 (-3.2, 18.0) | 2.20 | **.034** |
| -50ms vs -400ms | .089 (.023, .155) | 4.20 | **< .001** | 29.7 (8.1, 51.4) | 4.30 | **< .001** |
| -100ms vs -400ms | .104 (.045, .163) | 5.54 | **< .001** | 37.2 (17.5, 56.9) | 5.91 | **< .001** |
| -50ms vs -200ms | .070 (.016, .125) | 4.05 | **< .001** | 14.6 (.4, 28.9) | 3.21 | **.003** |
| -100ms vs -200ms | .085 (.035, .136) | 5.33 | **< .001** | 22.1 (10.0, 34.2) | 5.70 | **< .001** |
| -50ms vs 0ms | .053 (.008, .098) | 3.71 | **< .001** | 18.0 (9.0, 27.0) | 6.27 | **< .001** |
| -100ms vs 0ms | .068 (.017, .120) | 4.16 | **< .001** | 25.5 (13.4, 37.5) | 6.62 | **< .001** |
| -50ms vs +30ms | .100 (.038, .161) | 5.03 | **< .001** | 43.2 (25.9, 60.6) | 7.78 | **< .001** |
| -100ms vs +30ms | .115 (.057, .172) | 6.26 | **< .001** | 50.7 (30.9, 70.5) | 8.00 | **< .001** |
| -50ms vs +50ms | .102 (.038, .166) | 4.98 | **< .001** | 46.1 (24.2, 68.0) | 6.59 | **< .001** |
| -100ms vs +50ms | .117 (.060, .175) | 6.33 | **< .001** | 53.6 (32.4, 74.8) | 7.90 | **< .001** |
| -50ms vs +100ms | .131 (.073, .189) | 7.07 | **< .001** | 71.1 (54.7, 87.5) | 13.55 | **< .001** |
| -100ms vs +100ms | .146 (.093, .199) | 8.62 | **< .001** | 78.6 (61.7, 95.5) | 14.56 | **< .001** |
| -50ms vs +200ms | .138 (.083, .192) | 7.93 | **< .001** | 73.7 (59.7, 87.7) | 16.44 | **< .001** |
| -100ms vs +200ms | .153 (.102, .204) | 9.37 | **< .001** | 81.1 (69.9, 92.4) | 22.60 | **< .001** |

Note:
Confidence level used: 0.95

Benjamini-Hochberg adjusted for 15 tests

**Supplementary Table 3**

***Omnibus ANOVA Results for Experiment 3: Effects of Congruency × Location***

| Effect | Error Rate | | | | Response Time | | | |
| --- | --- | --- | --- | --- | --- | --- | --- | --- |
|  | *df* | *F* | *p* | η_p_^2^ | d*f* | *F* | *p* | η_p_^2^ |
| Congruency | 1, 21 | 41.03 | **< .001** | .66 | 1, 21 | 205.14 | **< .001** | .91 |
| Location | 1.5, 32.3 | 4.44 | **.028** | .17 | 1.5, 31.3 | 207.74 | **< .001** | .91 |
| Congruency × Location | 1.4, 29.8 | 5.98 | **.012** | .22 | 1.7, 35.4 | 31.38 | **< .001** | .60 |

Note:

Location and Congruency × Location effects corrected for sphericity using Greenhouse-Geisser method.
